# Lineage specific antigenic differences in porcine torovirus hemagglutinin-esterase (PToV-HE) protein

**DOI:** 10.1186/1297-9716-44-126

**Published:** 2013-12-23

**Authors:** Jaime Pignatelli, Julio Alonso-Padilla, Dolores Rodríguez

**Affiliations:** 1 Department of Molecular and Cellular Biology, Centro Nacional de Biotecnología, CSIC, C/Darwin 3, 28049 Madrid, Spain; 2 Current address: Centro de Investigación en Sanidad Animal (CISA-INIA), Carretera de Algete a El Casar km. 8.1, Valdeolmos, 28130 Madrid, Spain

## Abstract

Hemagglutinin-esterases (HE) are viral envelope proteins present in some members from the toro-, corona- and orthomyxovirus families, all related with enteric and/or respiratory tract infections. HE proteins mediate reversible binding to sialic acid receptor determinants, very abundant glycan residues in the enteric and respiratory tracts. The role of the HE protein during the torovirus infection cycle remains unknown, although it is believed to be important in the natural infection process. The phylogenetic analysis of HE coding sequences from porcine torovirus (PToV) field strains revealed the existence of two distinct HE lineages. In a previous study, PToV virus strains with HE proteins from the two lineages were found coexisting in a pig herd, and they were even obtained from the same animal at two consecutive sampling time points. In this work, we report antigenic differences between the two HE lineages, and discuss the possible implications that the coexistence of viruses belonging to both lineages might have on the spread and sustainment of PToV infection in the farms.

## Introduction

Toroviruses (ToV) are enveloped, positive single-stranded RNA viruses of cattle, horses, pigs and humans [1]. They have been associated with enteric infections and diarrhea, especially in young animals and children [2], and are considered a potential zoonotic threat [3]. The *Torovirinae* subfamily of the *Coronaviridae* family (order *Nidovirales*) comprises four species: equine torovirus (EToV), bovine torovirus (BToV), porcine torovirus (PToV) and human torovirus (HToV). The few epidemiological studies performed in different countries indicate high prevalences of these viruses [4-6]. The torovirus’s genome size (~ 28 kb) and organization are very similar to those of other coronaviruses, with two huge overlapping open reading frames (ORF) shaping the 5′-end of the genome, where the replicase/transcriptase machinery is encoded, and a final third of the RNA molecule hosting the coding sequences for the four structural proteins (from 5′ to 3′): spike (S), hemagglutinin-esterase (HE), membrane (M), and nucleocapsid (N) [7]. By analogy to other nidoviruses, the S protein is considered to be the putative receptor binding molecule. Copies of this molecule form the large spikes protruding from the viral particles as shown by electron microscopy [8,9]. The HE protein forms homodimers which make up the smaller spikes [5,10]. This HE protein is a class I membrane glycoprotein of about 65 kDa that belongs to the receptor destroying enzyme (RDE) protein family. As an RDE protein HE provides reversible binding to glycosylated surfaces [11] due to its ability to bind sialic acids and catalyze the disruption of that binding by means of its acetyl-esterase activity. The 3D structure and sialic acid preference of PToV- (strain Markelo) and BToV- (strain Breda) HE proteins have been solved [12]. Two main domains have been defined in the HE monomer of both proteins: the enzymatic acetyl-esterase region (E) and the receptor binding (R) or lectin domain.

Despite the great amount of knowledge acquired on this protein family, it is not yet clear what is the exact role of HE during the torovirus infectious cycle. The EToV, the only cell culture adapted torovirus, as well as different BToV strains that could be isolated in cell culture, all lack a functional HE protein [13-16] due to deletions or mutations in the HE gene, acquired during their adaptation to in vitro growth conditions. Thus, HE expression seems to be detrimental for in vitro culture of these viruses, as it occurs with the coronavirus murine hepatitis virus (MHV) [17]. However, the maintenance of the HE protein in a vast majority of new PToV and BToV field isolated strains indicates that it has to play an important function during in vivo infections. Several hypothetical functions have been postulated for the ToV-HE: (i) being a viral co-receptor, (ii) digestion of mucus layers to allow the virus to reach the target cells in the respiratory and/or enteric tracts, (iii) release of viral particles bound to decoy receptors, (iv) influencing host/cell tropism, or as recently proposed by de Groot’s group, (v) acting as a molecular timer in the virus pre-attachment step to the host-cell [18].

Two PToV-HE lineages have been identified, with representative strains being Markelo and P4 [19]. They share an amino acid sequence homology of 80%. Recently, during a longitudinal survey of PToV in a Spanish pig herd, several PToV-HE isolates representative of both lineages were identified [20], and similar findings were described from a survey performed in Korea [21]. In the first case, the two PToV-HE lineages were detected even within the same animal at two sequential sampling time points, indicating that both PToV strains carrying different HE proteins coexisted on the same farm infecting the same piglets, and suggesting that the immune response generated against one PToV strain did not protect the animals against the infection by the other strain. To further investigate this hypothesis PToV-HE proteins corresponding to each HE lineage were expressed, characterized, and used to track the anti-HE response in the animals from the farm where the PToV strains were obtained. The analysis of serum samples by hemagglutination inhibition assay and ELISA revealed antigenic differences between the two HE lineages.

## Materials and methods

### Cells, viruses and antisera

BSC40 (African green monkey kidney cells) cells were grown in Dulbecco’s modified Eagle’s medium (DMEM), supplemented with 5% heat-inactivated neonatal calf serum (NCS), non-essential amino acids (1%), gentamicin (50 μg/mL), penicillin (100 IU/mL), streptomycin (100 μg/mL) and fungizone (0.5 μg/mL).

Vaccinia virus (VACV), strain Western Reserve (WR) [22], and the recombinant VACV (rVV) derivatives were propagated and titrated in BSC40 cells.

To obtain a polyclonal antiserum against PToV-HE (αHE), a New Zealand rabbit was inoculated with two different peptides coupled to Keyhole limpet hemocyanin (KLH). Peptides comprising residues 51–67 (CTNPSTPNSLDIPQQLC) and 152–162 (LTPPENIPSHC) of PToV-HE-Bres [GenBank: FJ232070] [23] were chemically synthesized at the Proteomics facility of our institution after selection of target PToV-HE antigenic regions by bioinformatic analysis with Protean software (DNASTAR Inc. Madison, WI, USA).

### Pig serum samples

The serum sample collection and their treatment were previously described [20]. The samples corresponded to 12 animals from three different litters (A, B and C; four animals per litter) of the same farm, and were collected at 1-, 3-, 7-, 11-, and 15-weeks of age. Serum samples from the corresponding sow of each litter (*n* = 3) collected at the first day of sampling were also obtained.

### Sequence analysis and modeling

Sequences were retrieved from the NCBI database. The sequence analysis was performed by comparing an equal number of sequences related to each PToV lineage, obtained from viruses identified in three different geographical regions: Italy, Spain and Korea. Sequences representing the Markelo lineage were from the PToV strains Markelo [GenBank: GU299776.1], P78 [GenBank: AJ575367.1], 52.7 [GenBank: GU299776.1], 14.7 [GenBank: GU299775.1], 07-56-14 [GenBank: FJ555594.1] and 07-56-23 [GenBank: FJ555596.1]. The HE sequences representing the P4 lineage were from PToV strains P4 [GenBank: AJ575364.1], BRES [GenBank: FJ232070.1], 52.11 [GenBank: GU299777.1], 12.11 [GenBank: GU299773.1], 07-56-11 [GenBank: FJ555593.1] and 07-56-22 [GenBank: FJ555595.1]. Protein sequences were aligned using a ClustalW algorithm (MegAling, Lasergene, DNAstar, Inc).

Alignment modeling was performed on the Markelo HE structural model [PDB: 3I1K] using Pymol v1.1 (DeLano Scientific LLC). The amino acid residues that are conserved in all sequences from one lineage but are different from those in the strains corresponding to the other lineage are depicted in blue. Other residues that are conserved in all sequences or are non-lineage specific are depicted in red.

### Generation of rVV expressing PToV-HE genes

pGemT plasmids with PToV-HE coding sequences from isolates 52.7 and 52.11 [20] were used to sub-clone the indicated PToV-HE sequences into the pJR101 VACV transfer vector [24] by asymmetric digestion with restriction endonucleases *Bam*HI and *Nco*I. The resulting plasmids, pJR-HE52.7 and pJR-HE52.11, carried the HE genes under the control of the VACV synthetic early/late promoter (pe/L). Insertion of the HE genes into the VACV hemagglutinin locus (HA) was achieved by homologous recombination between the transfer vectors and the virus genome following standard procedures [25]. The resulting rVV-HE52.7 and rVV-HE52.11 viruses were selected and grown following standard procedures to yield viral stocks that were titrated and stored at −80 °C until use. A control rVV harboring a disrupted HA locus (rVV-HA^-^) was generated in parallel under the same procedures using the empty pJR101 vector.

rVV expressing soluble forms of HE52.7 and HE52.11 proteins fused to the c-myc tag were also generated to facilitate protein purification by affinity chromatography. For this purpose, the sequences coding for the fusion proteins were cloned into the pJR101 vector, and the corresponding rVV were obtained as described above.

### Western blot analysis

BSC40 cells infected with the corresponding rVV at a multiplicity of infection (MOI) of 5 plaque-forming units per cell (PFU/cell) were collected at 24 hours post-infection (hpi) in Laemmli sample buffer. Expression of PToV-HE proteins in infected cells was analyzed by polyacrylamide gel electrophoresis (SDS-PAGE) and Western blot with the rabbit polyclonal αHE serum. After incubation with a horseradish peroxidase-conjugated secondary antibody (Sigma-Aldrich, Saint-Louis, MO, USA), the reactive bands were detected by chemoluminescence using the commercial ECL reagent (GE Healthcare, Uppsala, Sweden).

### Immunofluorescence microscopy

Subconfluent BSC40 cell monolayers were grown in 12 mm-diameter coverslips and infected with rVV-HE52.7, rVV-HE52.11, or the control virus rVV-HA^-^ at an MOI of 5 PFU/cell. At 7 hpi, cells were washed, fixed with 4% paraformaldehyde and permeabilized with 0.5% Triton X-100 in PBS. After a blocking step with 20% fetal calf serum (FCS) in PBS, cells were incubated for 1 h with αHE polyclonal antiserum diluted (1:1000) in PBS containing 20% FCS, washed three times and stained with Alexa Fluor 594 goat anti-rabbit IgG (Molecular Probes™, Life Technologies, Carlsbad, CA, USA) at 1:500 dilution. DAPI reagent (4′, 6′-diamidino-2-phenylindole) (Molecular Probes™) was used to stain cell nuclei. After washing with PBS, coverslips were mounted on microscope slides using the ProLong® Gold anti-fade reagent from Molecular Probes™. Images were captured with a confocal Radiance 2100 system (Bio-Rad, München, Germany) and processed using ImageJ [26] and Adobe Photoshop CS4 (Adobe System Inc., San José, CA, USA).

### Cell extracts for HE functional assays

Confluent BSC40 cell monolayers seeded in 150 mm plates were infected with rVV-HE52.7 and rVV-HE52.11 at a MOI of 5. At 24 hpi cells were rinsed with PBS and harvested. After a centrifugation step at 3000 × rpm for 10 min, cell pellets were resuspended in TNE buffer (1 mM Tris-Cl, 75 mM EDTA and 25 mM NaCl) and subjected to three successive freeze-thaw cycles and sonication. The soluble fractions were stored at −80 °C until needed. To obtain esterase inactivated PToV-HE cell extracts, rVV infected BSC40 monolayers were treated with 5 mM diisopropyl fluorophosphate (DFP; Sigma-Aldrich) for 30 min at room temperature before scraping the cells. Any remaining excess of DFP was removed after thoroughly washing the cells with ice-cold PBS.

Protein concentrations in the cell extracts were determined by Bradford reaction (Bio-Rad Protein Assay) using known amounts of purified bovine serum albumin (BSA) as standards.

### Esterase assays

Specific acetyl-esterase activity of both recombinant HE proteins (HE52.7 and HE52.11) was tested by an in situ staining assay as previously described [27]. BSC40 cell monolayers were infected with the corresponding rVV-HE and with rVV-HA^-^ as the negative control. At 48 hpi viral plaques expressing HE were visualized after incubation with α-naphtyl acetate-Fast Blue BB solution (ANAE assay kit, Sigma-Aldrich) according to the manufacturer’s instructions. Cell monolayers were then stained with crystal violet solution (0.5% in 20% methanol) to visualize all viral plaques.

Sialate-*O*-acetylesterase activity of rVV-infected BSC40 cell extracts in TNE solution was determined with the synthetic substrate *p*-nitrophenyl acetate (*p*NPA; Sigma-Aldrich) as previously described [28]. Briefly, cell extracts were incubated with 3 mM *p*NPA at 37 °C, and the hydrolysis of *p*NPA was recorded by reading the absorbance at 405 nm at different times. The enzymatic activity per μg of cell lysate, after subtracting the background activity present in the control cell extract infected with rVV-HA^-^, was calculated as previously described [17].

### Hemagglutination and hemagglutination inhibition assays

These assays were performed in U-shaped 96-well plates (Nunc, Roskilde, Denmark). Mouse (*Mus musculus*, strain Swiss) blood obtained from the retro-orbital cavity of anesthetized animals was collected in two volumes of sterile Alsever solution to prevent clotting. Animals were handled following the guidelines of the Animal Experimentation Committee of our institution, in strict accordance with the Spanish law (RD 1201–2005). The red blood cells (RBC) obtained were washed with Alsever solution, counted and diluted in PBS to obtain a 10% stock solution (10% solution corresponds to 8 · 10^8^ RBCs per mL). Hemmagglutination assays (HA) were set-up using two-fold dilutions of extracts from DFP-treated and untreated rVV-infected cells. Fresh mouse RBC in PBS solution were added (50 μL) to the HE containing wells and incubated for two hours at 4 °C. Hemagglutination was scored and documented by photography, and plates were then placed at 37 °C and a second image was taken after 1 h incubation at this temperature. The hemagglutination units (HAU) of HE containing cell extracts were established as the reciprocal of the highest dilution causing hemagglutination.

For the hemagglutination inhibition assays (HI), HE cell extracts (4 HAU in 25 μL PBS) were incubated (1 h at 3 °C) with two-fold serial dilutions of kaolin treated pig sera [29] prior to adding 50 μL per well of a mouse erythrocyte suspension (final concentration of 0.5%). The HI titer was defined as the reciprocal of the highest serum dilution that completely inhibited hemagglutination by the viral antigen. Serum samples with HI titers higher than 3 log2 were considered positive.

### Purification of c-myc-tagged HE proteins

Extracts from BSC40 cells infected with rVV-HE52.7-myc or rVV-HE52.11-myc, were prepared as described above in TNE buffer containing 1% NP40. The soluble fractions were transferred to new tubes containing anti-c-myc coated beads previously equilibrated in TNE buffer (MBL, Naka-ku Nagoya, Japan). After 1 h incubation at 4 °C the mixtures of bead suspension and cell lysates were centrifuged in spin columns, washed thrice, and proteins were eluted with a c-myc tag peptide. The reagents and the protocol for the protein purification procedure were provided by the supplier. The purified proteins were analyzed by SDS-PAGE and Western blot with the αHE polyclonal serum and anti-c-myc monoclonal antibodies to confirm their identity (Clontech, Takara Bio Inc., Otsu, Shiga, Japan), and by Coomasie blue staining of the gel to determine their purity and concentration using different amounts of BSA as the reference.

### Enzyme-linked immunosorbent assay (ELISA)

An indirect ELISA was set up to detect antibodies against PToV-HE proteins in pig serum samples. The optimal protein concentration was established by checkerboard titration with positive and negative pig control sera. Paired rows of a 96-well microtiter plate (Maxisorp, Nunc) were coated overnight (4 °C) with purified HE52.7-myc and HE52.11-myc proteins diluted at a 1.25 μg/mL concentration. Plates were thoroughly washed after each step by rinsing the plates thrice with PBS containing 0.05% Tween 20 (PBST). After coating, plates were blocked for 2 h at 37 °C with PBST-3% BSA. Then, 50 μL of each serum sample diluted 1:100 in PBST-1% BSA was added in paired wells and incubated 1 h at 37 °C. A commercial goat anti-pig IgG secondary antibody conjugated to horseradish peroxidase (Sigma-Aldrich) was used. The enzymatic reaction was developed using *o*-phenylenediamine dihydrochloride (OPD-*FAST*™, Sigma-Aldrich), and stopped after 10 min by adding 50 μL/well of 2 N sulphuric acid. Optical densities at 492 nm (O.D._492_) were recorded with a multichannel spectrophotometer (Titertek Multiscan MCC/340). As negative control serum, a pool of sera from caesarian-derived, colostrum-deprived (CD/CD) pigs kept under germ free conditions (spf) was used, and the positive control serum was a commercial porcine serum (AbD Serotec, Kidlington, UK) previously determined to contain antibodies against PToV [23]. The ELISA cut-off was established for each antigen as the mean of the O.D. of negative control serum plus three times the standard deviation (HE52.7-myc cut off = 0.08; and HE52.11-myc cut off = 0.10).

A previously described indirect ELISA was used to detect antibodies to the highly conserved PToV nucleocapsid protein [23].

### Statistical analysis

Statistical analyses of HI and ELISA sera reactivities obtained from animals grouped by ages against both PToV-HE52.7 and PToV-HE52.11 were performed using two tailed *t* Student’s. Means ± standard error of the mean (SEM) for each group are shown.

## Results

### Expression of PToV-HE proteins

**Figure 1:**
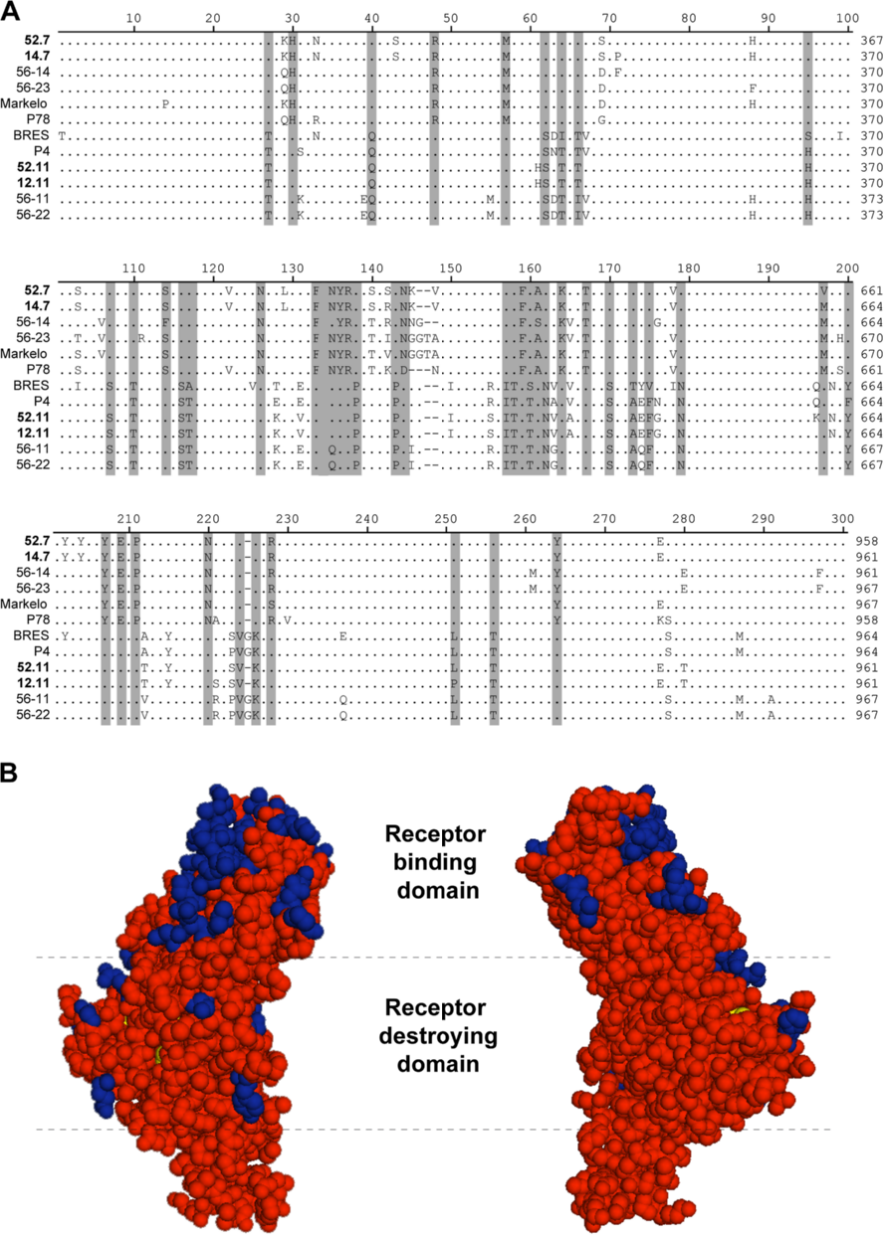
**Sequence analysis of HE proteins from PToV strains representative of both lineages. (A)** Multiple alignments of HE sequences. **(B)** Modeling over the Markelo HE structure (in red) of amino acid residues differing between Markelo and P4 lineages (in blue).

The detection of PToV strains belonging to the two defined HE lineages, represented by Markelo and P4 strains, in piglet fecal swabs collected on a Spanish farm in the course of a PToV longitudinal survey was previously described [20]. Interestingly, two PToV strains, one from each of the described lineages were isolated from the same animal (pig 52, litter B) at different time-points of the piglet’s life, at 7- and 11-weeks of age. The HE from the virus isolated at week 7, named PToV-HE52.7, was 94.4% homologous to Markelo HE at the amino acid level, whereas the protein from the virus found at week 11, HE52.11, was more closely related to P4 HE (93.4% homology). The direct comparison between both PToV-HE52.7 and PToV-HE52.11 gave a 77.9% identity, a homology that is similar to those obtained when comparing previous reported HE sequences from both HE lineages [19,21]. To study the amino acid differences between both HE lineages, a sequence alignment from HE proteins corresponding to viruses identified in three different geographical regions was performed. The HE sequences from the Spanish PToV strains HE12.11, HE52.11, HE14.7 and HE52.7 [20] were compared with those from other European strains Markelo, P78, P4 [19] and Bres [23], and from Korean strains 56–14 and 56–23 and 56–11 and 56–22 [21]. The alignment shows that several amino acid positions were conserved in a lineage specific manner (Figure 1A, grey shade), even in the geographically distant Korean strains. These lineage specific amino acids were located in the Markelo tridimensional structural model [12], and, as shown in Figure 1B, these differential amino acids (blue balls) were found mainly placed on the surface of the receptor binding domain, suggesting that they could mark potential antigenic differences between both HE lineages.

**Figure 2:**
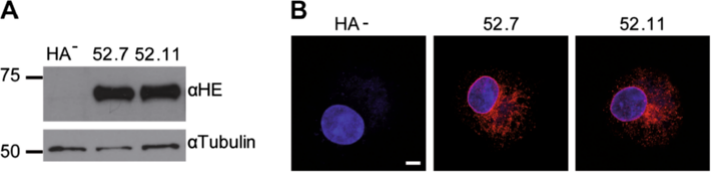
**Expression of PToV-HE52.7 and PToV-HE52.11 proteins. (A)** Western blot detection of HE proteins in extracts from BSC40 cells infected (MOI 5) with rVV-HE52.7, rVV-HE52.11 or rVV-HA-. Infected cells were fractionated by 10% SDS-PAGE, transferred to a nitrocellulose membrane and reacted with the αHE antibody. α-Tubulin signalling was taken along as a loading standard. Molecular size markers are given in kDa. **(B)** Immunofluorescence microscopy of BSC40 cells infected with rVV (MOI 5). Cells were fixed at 7 hpi, permeabilized and incubated with αHE antibody followed by a secondary antibody coupled to Alexa Fluor 594 (red). Cell nuclei were stained using DAPI (blue). Scale bar: 10 μm.

To study the HE proteins from PToV-HE isolates 52.7 and 52.11 as the model for the Markelo and P4 HE lineages, and search for potential antigenic differences between them, both proteins were expressed by the recombinant VACV methodology. rVV carrying the corresponding HE52.7 or HE52.11 coding sequences inserted into the HA locus of the VACV genome, were generated (rVV-HE52.7 and rVV-HE52.11). In addition, a VACV lacking a functional HA protein, rVV-HA^-^, was produced to serve as the negative control. PToV-HE proteins expressed upon infection of BSC40 cells with the rVV were specifically detected by Western blot and immunofluorescence microscopy using the αHE rabbit antiserum. Western blot analysis under denaturing conditions detected both HE52.7 and HE52.11 proteins in rVV-infected cell monolayers with the expected 65 kDa molecular weight (Figure 2A) corresponding to the glycosylated form of the protein [10]. Both HE proteins were expressed at a similar extent. Regarding the subcellular localization of recombinant HE proteins analyzed by immunofluorescence, the PToV-HE specific signal was widely distributed in the cytoplasm as well as associated to the nuclear and plasma membranes of BSC40 cells infected with either rVV-HE52.7 or rVV-HE52.11 but not in rVV-HA^-^- infected cells (Figure 2B).

### PToV-HE functional characterization

**Figure 3:**
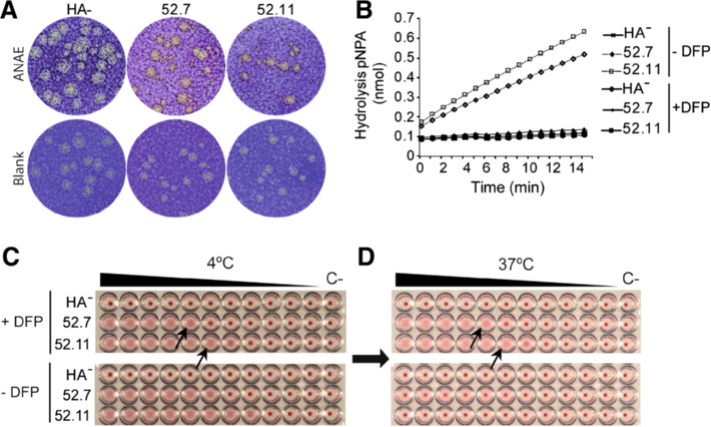
**Functional characterization of recombinant PToV-HE52.7 and PToV-HE52.11 proteins. (A)** ANAE in situ staining of rVV plaques produced in BSC40 infected with rVV-HE52.7, rVV-HE52.11 or rVV-HA- (~ 100 PFU per well). After the ANAE assay cell monolayers were stained with crystal violet to visualize all viral plaques. **(B)** Acetylesterase activity in extracts from BSC40 cells infected with rVV expressing PToV-HEs. Cells infected (MOI 5) with rVV-HE52.7 and rVV-HE52.11 or with the control virus rVV-HA- were harvested at 24 hpi, resuspended in TNE buffer and tested for acetyl esterase activity by incubation with 1 mM *p*NPA reagent. Background corrected hydrolysis of *p*NPA (in nmol), measured at 405 nm along a time span (min) is shown in the graph. **(C-D)** Hemagglutination assays with mouse RBC (0.5%) and 2-fold dilutions of both DFP-treated (+DFP) and untreated (−DFP) rVV-infected cell extracts resuspended in TNE buffer, recorded after incubation at 4 °C **(C)** and at 37 °C, **(D)**. The negative controls **(C-)** correspond to wells where RBC were incubated with TNE buffer.

The acetyl-esterase activity of the recombinant PToV-HE52.7 and HE52.11 proteins was analyzed by in situ staining of infected cells by the ANAE assay [27]. Infected cells surrounding viral plaques produced by both rVV-HE52.7 and rVV-HE52.11 show a brownish staining whereas the rVV-HA^-^ derived plaques remained unstained (Figure 3A). PToV-HE52.7 and rVV-HE52.11 were also able to hydrolyze the acetyl group from the synthetic substrate *p*-nitrophenol acetate in the *p*NPA assay [28] performed with extracts from infected cells. The graph in Figure 3B shows the specific *p*NPA hydrolysis obtained with the HE proteins. Both HE52.7 and HE52.11 cell extracts show similar activities (2.9 and 3.6 mU/μg lysate respectively). PToV-HE acetyl-esterase activity of the infected cell extracts was completely and irreversibly inhibited when the rVV-infected BSC40 cell monolayers were treated with the serine-esterase inhibitor DFP (see Materials and methods) as shown in Figure 3B. No esterase activity could be detected in extracts from cells infected with rVV-HA^-^, either untreated or treated with DFP.

The lectin activity of HE52.7 and HE52.11 proteins was tested by hemagglutination assay using mouse erythrocytes. The hemagglutination assay was set up with two fold serial dilutions of DFP treated (DFP^+^) and DFP untreated (DFP^-^) cell extracts, starting from 2 μg per well of total protein. The lysate from cells infected with rVV-HA^-^ served as the negative control. At 4 °C, both DFP^+^ and DFP^-^ rVV-HE cell extracts were able to induce hemagglutination of mouse RBC at a similar HA titer, whereas the HA^-^ cell extract did not hemagglutinate the mouse RBC at any of the amounts tested (Figure 3C). When plates were placed at 37 °C, the hemagglutination net induced by DFP^-^ cell extracts was disrupted due to their acetyl-esterase activity at that temperature, however hemagglutination was maintained in DFP-treated HE containing wells, indicating that the acetyl-esterase activity was completely inhibited (Figure 3D).

The esterase activity and receptor binding results show that both recombinant PToV-HE proteins were biologically active, indicating the acquisition of proper conformational folding.

### Inhibition of PToV-HE-induced hemagglutination by pig serum samples

**Figure 4:**
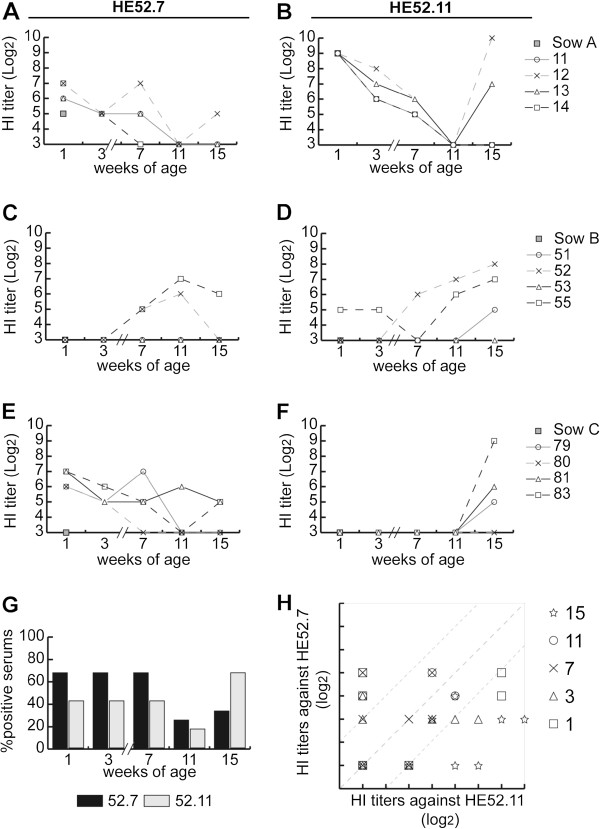
**Analysis by HI assay of the reactivity of sera obtained from pigs at different ages against PToV-HE52.7 and PToV-HE52.11.** Extracts from BSC40 cells infected (MOI 5) for 24 h with rVV-HE52.7 **(panels A, C and E)** or rVV-HE52.11 **(panels B, D and F)** and treated with DFP were diluted to contain 4 HAU in 25 μL PBS and incubated (1 h at 37 °C) with two-fold serial dilutions of pig sera prior to adding the mouse RBC. Serum samples were obtained from animals belonging to three litters **(litter A, panels A and B; litter B, panels C and D; litter C; panels E and F)** at different ages (1, 3, 7, 11 and 15 weeks) and from their sow (one-week post-farrowing). **(G)** Percentage of HI positive animals against PToV-HE52.7 (blacK bars) and PToV-HE52.11 (grey bars) over the period studied. **(H)** Comparative analysis of HI titers of each serum sample against PToV-HE52.7 and PToV-HE52.11 proteins. The results shown in the graphs are representative of two independent experiments.

The hemagglutination caused by HE proteins is due to the binding of their lectin or receptor binding domain to specific sialic acid determinants present on the surface of RBC. Hence, in order to examine the potential existence of antigenic differences between the two lineages of PToV-HE proteins, piglet serum samples were analyzed with an HI assay to detect the presence of specific antibodies against the receptor binding domain that prevents the RBC hemagglutination. In the HI test, DFP inactivated HE52.7 and HE52.11 cell lysates were used to avoid artifacts in the assay derived from the HE esterase activity. Using serum samples from the longitudinal survey, different HI antibody response dynamics against HE52.7 and HE52.11 proteins were observed. Anti-HE52.7 antibodies were detected at week 1 in 65% of the piglets (7/12) (Figure 4G), even though all piglets from litter B were negative against that particular lineage at that time-point (Figure 4C). With respect to the anti-HE52.11 inhibitory reaction in week 1 sera, none of the animals in litter C was reactive against it (Figure 4F), and only one piglet from litter B had specific anti-HE52.11 HI reactivity (Figure 4D). However all piglets from litter A had high HI titers towards this lineage, in agreement with the high HI titer of their sow (Figure 4B). Independently of their lineage specificity, the HI titers decreased at week 3 (coinciding with weaning) in all but one anti-HE positive piglets, however, the percentages of positive animals against both HE proteins remained constant until week 7 (Figure 4G). Up to that week, the reactivity to HE52.11 was circumscribed to piglets within litter A (Figure 4B). At week 11, just 25% of the piglets (3/12) were reactive against HE52.7, and a bare 20% presented anti-HE52.11 antibodies (Figure 4G). By week 15, 67% of the piglets (8/12) were positive against HE52.11 (Figure 4G). Although anti-HE52.7 reactivity was also observed in 33% (4/12) of the piglets, all those animals had higher HI titers against HE52.11.

To compare the reactivity of each serum sample against both HE52.7 and HE52.11 proteins the HI titers obtained against the two HE proteins were plotted in Figure 4H. While few samples had the same HI titers with both proteins (serum samples found over the diagonal), most of them showed preferential reactivity with one versus the other HE lineage (serum samples found at both sides of the diagonal) and even some serum samples showed specific reactivity against only one of the HE proteins (7 serum samples were positive only for the HE52.7 and 5 were specific for the HE52.11). These data clearly indicate that HE lineage specific amino acid differences within the receptor domain were enough to determine that antibodies developed against one lineage do not interfere with the receptor recognition by the other HE lineage.

### Analysis of the antibody response to HE in pigs by ELISA

To further investigate the reactivity of antibodies in piglets’ serum samples against HE52.7 and HE52.11 proteins and the dynamics of antibodies against each of them, an ELISA method using purified myc-tagged HE proteins (HE52.7-myc and HE52.11-myc) as antigens was used. The analysis by SDS-PAGE confirmed the purity of both preparations and their reactivities with both αHE polyclonal serum and anti-c-myc monoclonal antibodies were confirmed by Western blot (see Additional file 1).

**Figure 5:**
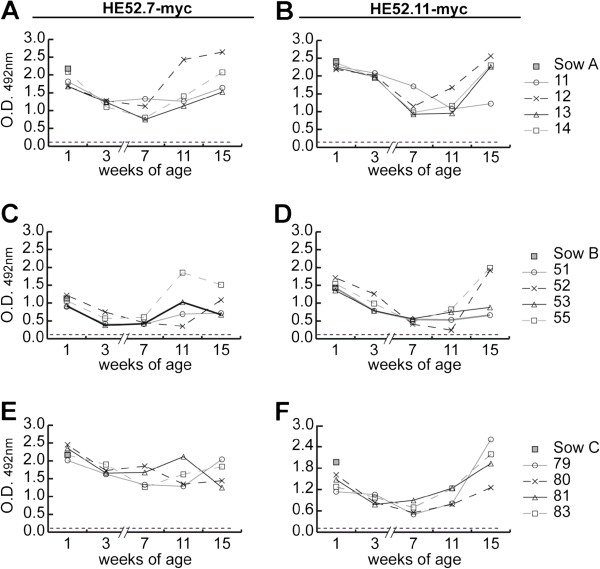
**Reactivity of sera obtained from pigs at different ages against PToV-HE52.7 and PToV-HE52.11 determined by ELISA.** Purified HE52.7-myc- and HE52.11-myc proteins were used as coating antigen (62.5 ng/well). The same serum samples analyzed in Figure 4 were used in the ELISA at a 1:100 dilution. IgG ELISA reactivities against HE52.7-myc- **(panels A, C and E)** and HE52.11-myc **(panels B, D and F)** of serum samples from pigs and their sows are represented.

All pig serum samples tested were positive against both HE proteins by ELISA (Figure 5). One-week-old piglets had O.D. values akin to those of their corresponding sow against each HE protein. Piglets from litter A were highly reactive against both HE52.7 and HE52.11, although the ELISA values were slightly higher against the second one (Figure 5A and 5B). Piglets from litter B, had a low reactivity against both proteins (Figure 5C and 5D). In contrast, piglets from litter C showed a higher reactivity against HE52.7 than against HE52.11 (Figure 5E and 5F). As it happened with HI titers, anti-HE reactivity by ELISA gradually diminished with piglet’s age and by 7 weeks of age, 4 weeks after weaning, it reached the lowest level in most of the animals. At the next age analyzed (11 weeks), most animals showed an increase in their anti-HE reactivities against both PToV-HE proteins, and these kept rising until 15 weeks of age at least.

**Figure 6:**
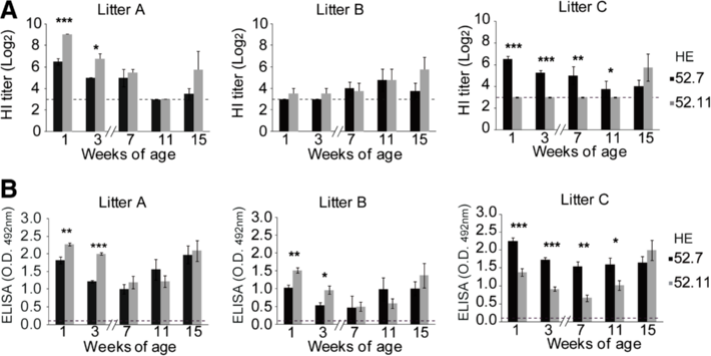
**Paired comparisons of the results of pig sera reactivities against PToV-HE52.7 and PToV-HE52.11 determined by HI and ELISA.** Graphic representation of the mean HI **(A)** and ELISA **(B)** titers of sera grouped by pig ages against both PToV-HE52.7 (black bars) and PToV-HE52.11 (grey bars). Means ± standard error for each sample are shown. Asterisks indicate statistically significant differences of sera reactivity against the two proteins (**p < 0.05, *p < 0.1; Student’s *t* test). The dotted lines indicate the respective cut off values determined for each assay.

To obtain an overview of the immune response against both PToV-HE proteins, the mean ELISA reactivities and HI titers of the serum samples from each litter grouped by ages were compared side-by-side. As shown in Figure 6, both assays revealed statistically significant antigenic differences between HE52.7 and HE52.11. In animals from litters A and B, higher reactivity against HE52.11 was observed in the first weeks of the piglets’ life, although low titers were observed in animals from litter B, which were barely detectable by the HI assay. On the contrary, in the first weeks, animals from litter C had higher antibody titers against HE52.7 by both assays. Also, from these results it is clear that sera from 15-week old animals from the three litters reacted preferentially against HE52.11, indicating a switch in antibody reactivity in animals from litter C.

### Antibody response to the PToV-N protein

The reactivity of the piglet’s sera against the highly conserved N protein using a previously standardized ELISA [23] has already been analyzed [20]. For comparison with the anti-HE immune response here we provide the results of the analysis of sera from the individual piglets over time (see Additional file 2). Although there are differences in the magnitude of the ELISA titers among the different piglets, what is clear from these results is that once maternally acquired antibodies had vanished, which for the N protein occurs around weaning time (week 3 of age), all animals developed their own anti-N antibodies that can readily be detected in most animals by week 7. These findings were in agreement with those obtained by HI and ELISA using the HE proteins as antigen, and indicate that all animals become infected by PToV soon after weaning.

## Discussion

Despite the great advances recently made on HE knowledge, which include the elucidation of both BToV-HE and PToV-HE tridimensional structures [12], the exact role of the HE protein in the viral life cycle and the potential relevance of the differences between HE lineages in the immunological response to the virus remain unclear. Without an in vitro culture system and given the great difficulties and costs of in vivo research, the use of heterologous expression systems represents a useful and relatively inexpensive approach to study the PToV-HE protein. Here we used the recombinant VACV methodology to express two full PToV-HE sequences, HE52.7 and HE52.11, and their two corresponding soluble fractions attached to a c-myc tag. Those HE coding sequences had been previously identified during a thorough longitudinal study of PToV in a Spanish farm [20], and each of them were found to belong to one of the two defined PToV-HE lineages [19]. These HE sequences were obtained from the same animal in sequential collection points. This finding indicated that both lineages co-existed on the farm [20] although with apparently different prevalences according to the age of the host. This result could lead to new possibilities to approach the PToV-HE behavior in the virus’ natural environment.

Both recombinant proteins show similar features regarding their molecular weight, glycosylation degree, and subcellular localization as those reported previously for PToV and BToV [10,17], indicating that the recombinant HE proteins follow a correct biosynthetic pathway. In addition, the heterologous expressed HE proteins were fully functional as receptor binding-receptor destroying enzymes since both PToV-HE proteins were able to hemagglutinate mouse erythrocytes, but also to hydrolyze the acetyl-ester linkage of glycan chains, as well as from acetylated synthetic compounds like *p*NPA and ANAE.

The analysis of the amino acid changes found between both PToV-HE lineages shows that there are amino acid residues that are conserved in a lineage specific manner even among strains identified in very distant geographic areas (different European countries and Korea). This analysis also indicates that potential antigenic differences would be mainly determined by residues located at the receptor binding domain, and exposed on the surface of the protein according to the proposed structural model [12]. Hence, to elucidate if HE lineage specific changes could determine antigenic differences on the receptor binding domain, the HE 52.7 and 52.11 proteins were used as model proteins in HI assays with field serum samples from the same farm where both lineages were detected. Significantly, by this assay most serum samples show preferential reactivity to one of the HE proteins, or even specific reactivity against only one of them (see Figure 4H), indicating the existence of antigenic differences between the two HE lineages. Antigenic differences between HE proteins from the two known BToV lineages have also been described [17].

In addition, to study the antibody response against the whole protein by a different approach, soluble c-myc tagged HE52.7 and HE52.11 proteins were generated by rVV methodology to obtain highly purified coating antigens that were used in ELISA to test the same field serum samples. Using both approaches, a high prevalence of antibodies against PToV-HE was observed in both sows and piglets. These results were in agreement with those obtained using an ELISA assay with the very immunogenic and highly conserved N protein as antigen [23].

In the present study, similar anti-HE response profiles over time were observed by both HE-ELISA and the more restricted lectin-specific HI test. A general decrease of antibody levels was seen from the first weeks of age until piglets’ reached week 7, related to the extinction of maternally derived initial immunoglobulins. At 11-weeks of age, immune levels recovered due to the development of the pigs’ own response to infections, and they increased at least until week 15. At this last sampled point, a general increase of reactivity against HE52.11 (P4-like) was found in animal sera from the three litters, indicative of the prevalence of this lineage upon time. Overall, similar antibody patterns were observed by ELISA against the HE proteins and the N protein, although a delay in both the extinction of maternally derived antibodies and the development of self-acquired antibodies against the HE was observed in all animals analyzed (see Figure 5 and Additional file 2).

The rising of antibodies to PToV antigens after weaning can be explained as a consequence of the animal grouping in livestock facilities for fattening purposes that provides the conditions for piglet infection and/or re-infection and the mixture of PToV strains in the same animal population. Our results indicate that the immune response developed against one of the PToV lineages could not protect against the infection by other PToV isolates carrying an HE protein belonging to a different lineage. Hence, the specificity of the piglet’s current immune response, its own or maternal, towards one or the other HE lineage at a given time could determine the PToV strain that could infect or prevail in the animal. Our hypothesis is that this PToV lineage alternation could explain the sustainment of both strains on the farm. In fact, even though the P4-like (HE52.11) strain was not found by molecular detection in piglets at the earliest ages [20], the high reactivity observed by ELISA in sows and young piglets and, more remarkably, by the HI in sow A and in 1-, 3- and 7-week old piglets from litter A meant that such a strain was already circulating in the herd from the very beginning of the sampling. Particularly, in piglet 52 the increasing reactivity against both HE proteins from week 3 to week 15 indicates a temporal coexistence of both types of virus in the piglet at around weaning time. The lack of anti-HE maternal antibodies with an HI capacity against either HE protein might have facilitated the establishment of both PToV strains early on in the piglet’s life. Although we described that the two PToV-HE lineages were detected within the same animal at two sequential sampling time points, the lack of detection of PToV-HE52.11 at week 7 could have been due to the low abundance of this virus at the beginning of life, while at later times it became predominant, and therefore easier to detect. Although the shift from PToV-HE52.7 to PToV-HE52.11 in the analyzed animals (Markelo-like to P4-like strains) seems to derive from immune pressure on the latter, the contribution of a potential better viral fitness provided by the former on older pigs’ tissue environments cannot be discarded.

The tendency to recombine modules of their genomes observed in *Nidovirales*, together with the extensive mutation rates found, like in other RNA viruses, would facilitate the evasion of immune responses and the rapid adaptation to new hosts and the new hosts’ environments. From this study, where two PToV HE genotypes were found co-existing on the same farm, we can speculate that the specificity of the immune response towards one or the other HE lineage in piglets at a given time could determine the PToV strain that prevailed and spread. However, the potential additional contribution of the immune response to other viral antigens in virus selection also has to be considered. Future studies with higher numbers of animals from different farms will be required to further support the proposed hypothesis.

Though the PToV-HE protein in vivo function/s are still to be undoubtedly defined, its persistence in field strains, the tendency to undergo recombination events and the different antigenic characteristics of both HE lineages indicate that HE protein from torovirus plays an important role in virus–host interactions with implications in immune protection that could explain the broad spread of this virus in the pig population, causing chronic infections/re-infections of the animals.

## Competing interests

The authors declare that they have no competing interests.

## Authors’ contributions

JP and JAP have contributed equally to this work. JP and JAP participated in the design of the study, generated recombinant viruses to express recombinant HE proteins, contributed to characterize the recombinant HE proteins, carried out HI assays, participated in the analysis of the results and draft of the manuscript. DR conceived the study, participated in its design, coordination and draft of the manuscript. All authors read and approved the final manuscript

## Supplementary Material

Additional file 1 **Analysis of purified PToV-HE52.7-myc and PToV-HE52.11-myc proteins.** (A) Cell extract from BSC40 cells infected (MOI 5) with rVV-HE52.7-myc or rVV-HE52.11-myc (lysate), and affinity purified HE52.7-myc and HE52.11-myc proteins (protein) were fractionated by 10% SDS-PAGE, and the gel was stained with Coomassie blue. (B) Affinity purified HE52.7-myc and HE52.11-myc proteins were reacted in Western blot with the αmyc and αHE antibodies. Molecular size markers are given in kDa. Click here for file

Additional file 2 **Reactivity of sera obtained from pigs at different ages against the N protein.** The same serum samples analyzed in Figure 4 were used in ELISA at a 1:100 dilution using the PToV-N protein as antigen as previously described [23]. Click here for file
