# Supplementary figures and images for: Lineage specific antigenic differences in porcine torovirus hemagglutinin-esterase (PToV-HE) protein
Source: Vet Res. 2013 Dec 23;44(1):126. doi: 10.1186/1297-9716-44-126 (PMC3878402; doi:10.1186/1297-9716-44-126)

**A)**

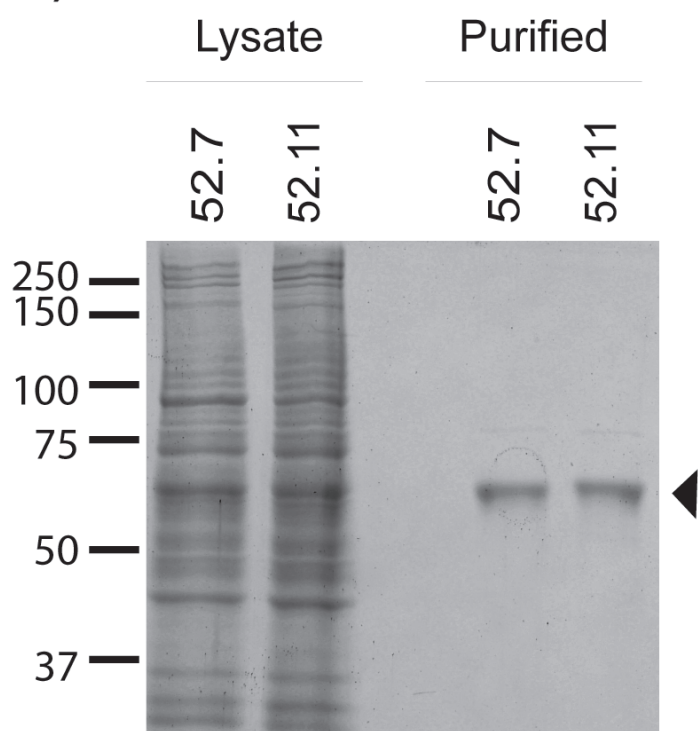

**B)**

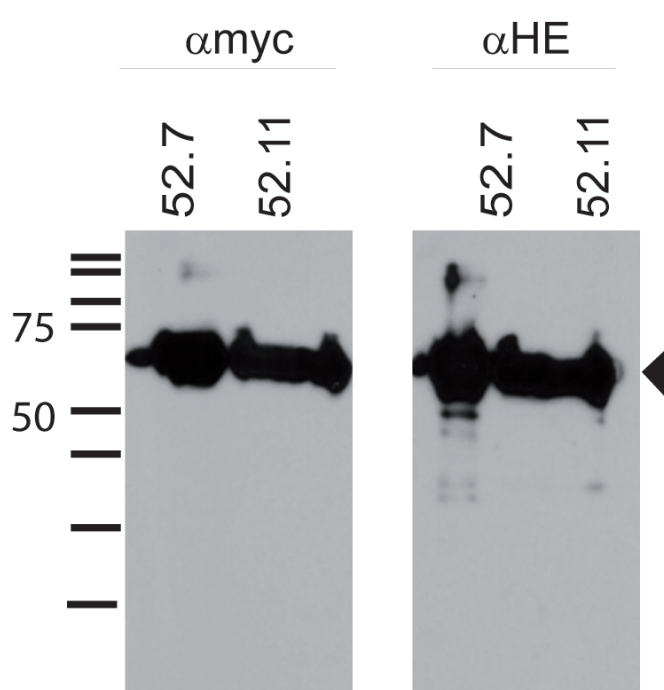

Supplement: Additional file 1 — Analysis of purified PToV-HE52.7-myc and PToV-HE52.11-myc proteins. (A) Cell extract from BSC40 cells infected (MOI 5) with rVV-HE52.7-myc or rVV-HE52.11-myc (lysate), and affinity purified HE52.7-myc and HE52.11-myc proteins (protein) were fractionated by 10% SDS-PAGE, and the gel was stained with Coomassie blue. (B) Affinity purified HE52.7-myc and HE52.11-myc proteins were reacted in Western blot with the αmyc and αHE antibodies. Molecular size markers are given in kDa. [file 1297-9716-44-126-S1.pdf]

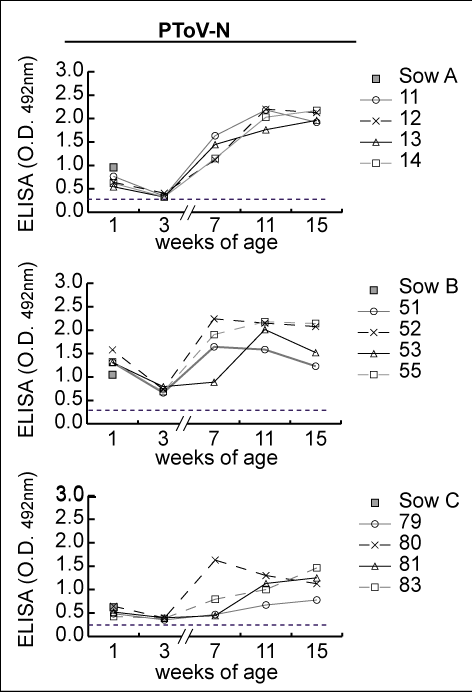

Supplement: Additional file 2 — Reactivity of sera obtained from pigs at different ages against the N protein. The same serum samples analyzed in Figure 4 were used in ELISA at a 1:100 dilution using the PToV-N protein as antigen as previously described [23]. [file 1297-9716-44-126-S2.tiff]
